# Supplementary material for: The genome sequence of Erebia montana Berg-Mohrenfalter (Lepidoptera: Nymphalidae)
Source: Wellcome Open Res. 2025 Aug 18;10:449. [Version 1] doi: 10.12688/wellcomeopenres.24675.1 (PMC12648014; doi:10.12688/wellcomeopenres.24675.1)
Supplement: Peer review history

# Reviewer report: Reviewer response for version 1

**DOI:** 10.21956/wellcomeopenres.27190.r138401

**Published:** 2025-11-27

**Authors:** Li-Wei Wu

**Affiliations:** 1 Tunghai University, Taichung, Taichung City, Taiwan

This article provides genomic information for a female specimen of the nymphalid butterfly *Erebia montana*, and I believe it will serve as a valuable reference for subsequent research. Overall, the presentation of data is consistent with established genome-generation procedures. However, on page 3, within the section “Nucleic acid extraction,” the phrase “a yield of 1 934.52 ng” appears to contain an unintended space between the digits “1” and “9.” I am uncertain whether this spacing is purposeful or accidental, but it likely requires correction. In addition, this study reports two distinct genomic assemblies derived from the same female individual. I feel that further clarification of this aspect would be important. For example, whether this reflects potential chromosome-level chimerism, or whether it results from substantial heterozygosity between haplotypes. It may also be helpful to characterize the differences between the two genome haplotypes using the currently available assemblies. As the researchers who directly conducted the wet-lab assembly work, the authors are in the best position to clarify these points, and I believe that explicitly addressing this issue would greatly benefit researchers who may rely on this genome in future comparative or evolutionary studies.

Are sufficient details of methods and materials provided to allow replication by others?

Yes

Is the rationale for creating the dataset(s) clearly described?

Yes

Are the datasets clearly presented in a useable and accessible format?

Yes

Are the protocols appropriate and is the work technically sound?

Yes

Reviewer Expertise:

butterfly conservation, phylogeography

I confirm that I have read this submission and believe that I have an appropriate level of expertise to confirm that it is of an acceptable scientific standard, however I have significant reservations, as outlined above.

# Reviewer report: Reviewer response for version 1

**DOI:** 10.21956/wellcomeopenres.27190.r139222

**Published:** 2025-11-25

**Authors:** Jasmine D Alqassar

**Affiliations:** 1 The George Washington UniversityWashington, District of Columbia, USA

In this article Lucek et al. present a high-quality chromosome-level reference genome for Erebia montana. BUSCO analysis demonstrates the genome assembly is highly contiguous and complete (BUSCO score: C: 98.6%[S:94.6%‚D:4%]‚ F:0.3%‚ M:1.1%‚ n:5286). Additionally, this assembly is chromosome-level with 98.25% of the assembly assigned to chromosomes. This quality chromosome-level genome resource will serve as a valuable resource for the lepidopteran genomics community. The methods are well-explained and the genome resource is already accessible to the public. The inclusion of Merian element assignment will serve as an important resource for chromosome evolution studies.

 The addition of an explanation for why scaffold OZ178013.1 is termed as a Z2 sex chromosome as well as a statement that compares this finding to existing Erebiini genomes will strengthen this article.

Are sufficient details of methods and materials provided to allow replication by others?

Yes

Is the rationale for creating the dataset(s) clearly described?

Yes

Are the datasets clearly presented in a useable and accessible format?

Yes

Are the protocols appropriate and is the work technically sound?

Yes

Reviewer Expertise:

Genomics and evolutionary developmental biology

I confirm that I have read this submission and believe that I have an appropriate level of expertise to confirm that it is of an acceptable scientific standard.

# Reviewer report: Reviewer response for version 1

**DOI:** 10.21956/wellcomeopenres.27190.r138403

**Published:** 2025-11-10

**Authors:** Yang Mei

**Affiliations:** 1 Zhejiang University, Hangzhou, China; 2 Jilin Agricultural University (Ringgold ID: 85112), Changchun, Jilin, China

The authors reported a high-quality genome of *Erebia montana* Berg-Mohrenfalter (Lepidoptera: Nymphalidae). But I have some questions:

 1. Why is there a difference of 100MB betweenthe  two haplotypes? The difference is so significant

 2. The Z chromosome is very conserved in Lepidoptera and is often the largest chromosome. Why was it not fully assembled in this study

 3. Maybe providing the genome annotation is well

Are sufficient details of methods and materials provided to allow replication by others?

Yes

Is the rationale for creating the dataset(s) clearly described?

Yes

Are the datasets clearly presented in a useable and accessible format?

Yes

Are the protocols appropriate and is the work technically sound?

Yes

Reviewer Expertise:

insect genomcis

I confirm that I have read this submission and believe that I have an appropriate level of expertise to confirm that it is of an acceptable scientific standard.
